# Synthesis of ZnO Nanocrystals and Application in Inverted Polymer Solar Cells

**DOI:** 10.1186/s11671-017-2283-6

**Published:** 2017-09-09

**Authors:** Jing-Jing Dong, Jian Wu, Hui-Ying Hao, Jie Xing, Hao Liu, Hua Gao

**Affiliations:** School of Science, China University of Geosciences, Beijing, 100083 China

**Keywords:** Zinc oxide, Nanocrystals, Hydrothermal, Solar cells, 73.61.Ga, 73.61.Tm, 81.10.Dn, 84.60.Jt

## Abstract

Controllable synthesis of various ZnO nanocrystals was achieved via a simple and cost-effective hydrothermal process. The morphology evolution of the ZnO nanostructures was well monitored by tuning hydrothermal growth parameters, such as solution concentration, reaction temperature, and surfactant. As-obtained ZnO nanocrystals with different morphologies, e.g., ZnO nanorods, nanotetrapods, nanoflowers, and nanocubes, were further introduced into the organic bulk heterojunction solar cells as the electron transport channel. It was found that the device performance was closely related to the morphology of the ZnO nanocrystals.

## Background

Organic bulk heterojunction solar cells using n-type inorganic metal oxide nanostructures as the electron transport channel have attracted considerable attentions because of its improved ambient device stability, low-cost manufacturing, and compatibility to solution fabrication process [1–4]. ZnO nanocrystals, which have high electron mobility, excellent stability, good transparency in the whole visible range, simple preparation process, and easier tailoring of the nanostructures, are promising candidates as the electron transport channel in organic bulk heterojuction solar cells. Recently, various ZnO nanostructures, e.g., nanorods, nanowalls, and nanotetrapods, have been introduced to the organic bulk heterojunction solar cells [5–7]. And it is reported that the device performance is improved by providing a short and continuous pathway for electron transport, enhancing the exciton dissociation ratio, or increasing the ZnO/active layer interface area. However, the relationship between the morphology of the ZnO nanocrystals and the device performance is still controversial.

In this paper, we prepared ZnO nanocrystals with different morphologies via a simple and cost-effective hydrothermal process. The morphology of the ZnO nanostructures was tuned effectively by varying hydrothermal growth parameters, such as the solution concentration, reaction temperature, and surfactant. As-obtained ZnO nanocrystals with different morphologies, e.g., ZnO nanorods, nanotetrapods, nanoflowers, and nanocubes, were introduced into the organic light absorber as the electron transport channel. The current density-voltage (*J*-*V*) result reveals that the device performance is closely related to the morphology of the ZnO nanocrystals. To improve the device performance, large surface area and proper space between adjacent ZnO nanocrystals for the infiltration of the organic light absorber, as well as a short and continuous pathway for electron transport, are essential.

## Methods

### Deposition of ZnO Seed Layer

To grow ZnO nanocrystals on mismatched substrates, the ZnO seed layer is essential. In this paper, the ZnO seed layer is prepared by the dip-coating method, which has been described in our previous paper [8].

### Hydrothermal Growth of ZnO Nanocrystals

To grow various ZnO nanostructures, the indium-tin-oxide (ITO) substrate coated with the ZnO seed layer was fixed upside down in the reaction vessel filled with 40 ml aqueous solution of zinc nitrate hexahydrate (Zn(NO_3_)_2_·6H_2_O) and hexamethylenetetramine (HMTA) with the identical concentration. Then, a certain amount of surfactant, such as polyethylenimine (PEI) or sodium citrate, was added in the aqueous solution [8]. Next, the reaction vessel was sealed and kept at a constant temperature for a certain time. Finally, as-grown ZnO nanocrystal was taken out, rinsed in deionized water, and dried in air for use.

### Fabrication of the Solar Cells [9]

**Fig. 1:**
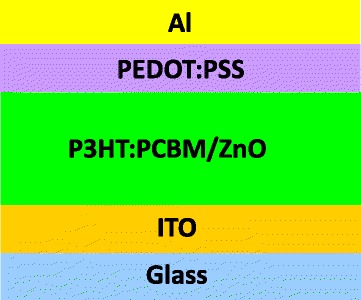
Device architecture of the organic bulk heterojunction solar cell

Firstly, a thin PCBM layer was spin-coated onto the ZnO nanocrystal from a dichloromethane solution with the concentration of 20 mg/ml, at 1000 rpm for 30 s. It was reported that the [6]-phenyl-C61-butyric acid methyl ester (PCBM) layer between the ZnO nanocrystal and organic active layer could improve the infiltration of the active polymer layer into the gaps of ZnO nanocrystals [10]. Then, the active layer comprising of poly(3-hexylthiophene) (P3HT, 10 mg/ml) and PCBM (16 mg/ml) blending in chlorobenzene was spin-coated on the top of the PCBM layer at 1000 rpm for 30 s. After that, the samples were baked at 225 °C for 1 min to remove the residual solvent and assist the polymer infiltrate into the gaps of ZnO nanocrystals. Next, poly(3,4-ethylene dioxythiophene):poly(styrene sulfonate) (PEDOT:PSS) hole transport layer was spin-coated at 4000 rpm for 40 s, and then thermally annealed at 130 °C for 15 min in air, resulting in a ~ 35-nm thick PEDOT:PSS layer. Finally, 100 nm Al was deposited by thermal evaporation as a cathode to create a device. Finally, the devices were thermally annealed at 130 °C for 20 min on the hotplate under nitrogen ambient. The final device structure is shown in Fig. 1.

### Characterization

Surface morphologies of the ZnO nanocrystals were characterized by field emission scanning electron microscopy (SEM; FE-S4800, Hitachi, Tokyo, Japan). The *J*-*V* characteristics of solar cells were taken using a Keithley 2400 source measure unit under 100 mW/cm^2^ illumination (AM 1.5G).

## Results and Discussion

**Fig. 2:**
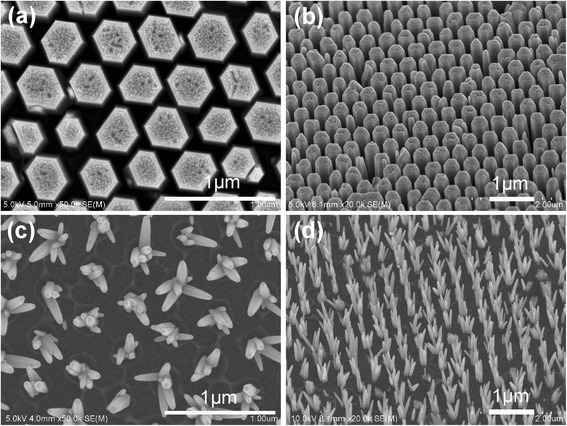
**a** Top view and **b** 45° tilt view of the ZnO nanorod array. **c** Top view and **d** 45° tilt view of the ZnO nanotetrapod array

By tuning hydrothermal growth parameters, such as the solution concentration, reaction temperature, and surfactant, ZnO nanocrystals with different morphologies, e.g., ZnO nanorods, nanotetrapods, nanoflowers, and nanocubes, were obtained. Among them, patterned and aligned ZnO nanorod array was synthesized via a hydrothermal route by using the TiO_2_ ring template deriving from the polystyrene microsphere self-assembled monolayer (inverted self-assembled monolayer template), which has been demonstrated in our previous work [11]. Figure 2a, b presents the top and 45° tilt view of the as-grown ZnO nanorod array, grown in the aqueous solution containing 0.05 M Zn(NO_3_)_2_·6H_2_O and HMTA at 80 °C for 3 h. It can be seen that the ZnO nanorod array reserves the long-range hexagonal periodicity of the TiO_2_ ring template very well. All of the ZnO nanorods are perfectly aligned normal to the substrate with the uniform diameter of 380 nm, which can provide a short and continuous pathway for electron transport, and only one ZnO nanorod is grown at each growth site. From the top view of the as-grown ZnO nanorod array in Fig. 2a, we can see that the space between adjacent ZnO nanorods is about 200 nm wide, which is important for the following infiltration of the organic light absorber. Besides, both the diameter and length of the ZnO nanorods can be varied easily by varying the solution concentration and reaction temperature during hydrothermal growth, as reported in our previous work [11]. The ZnO nanotetrapod array, as shown in Fig. 2c, d, was grown at 0.025 M, 50 °C for 6 h by the inverted self-assembled monolayer template similar with the ZnO nanorod array. The difference from the ZnO nanorod array is that a certain amount of PEI (0.1 ml PEI per 40 ml reaction solution) has been used during the hydrothermal growth, which is reported to promote the growth in the axial direction, but suppress the growth in the radial direction [12]. From the top view (Fig. 2c) and 45° tilt view (Fig. 2d) of the ZnO nanotetrapod array, we can see that the nanotetrapod array also reserves the long-range hexagonal periodicity of the TiO_2_ ring template very well, and each nanotetrapod is composed of three to seven nanorods grown at each growth site, so the surface area of the ZnO nanotetrapod array is much larger than the ZnO nanorod array.

**Fig. 3:**
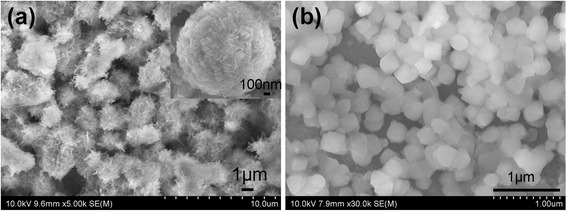
Top view of **a** the ZnO nanoflowers and **b** the ZnO nanocubes. The inset of Fig. 3a is the zoom-in view of a single ZnO nanoflower

Figure 3a, b shows the SEM images of ZnO nanoflowers and nanocubes, respectively, which are prepared by a two-step method, as follows. Firstly, ZnO nanorods were grown via the hydrothermal process in the aqueous solution containing 0.025 M Zn(NO_3_)_2_·6H_2_O and HMTA at 85 °C for 3 h. Then, as-grown ZnO nanorods were immersed into different solutions for secondary growth. ZnO nanoflowers were obtained in the solution of 0.0075 M Zn(NO_3_)_2_·6H_2_O and 0.0075 M sodium citrate at 95 °C for 12 h, while ZnO nanocubes were obtained in the solution of 0.0075 M Zn(NO_3_)_2_·6H_2_O and 0.015 M sodium citrate at 95 °C for 6 h. Finally, as-grown ZnO nanoflowers and nanocubes were thoroughly rinsed with deionized water and dried in air to remove residual polymer. From the top view of the ZnO nanoflowers in Fig. 3a, we can see that the ZnO nanoflowers are unordered and crowded, and each nanoflower is composed of many “petals”, so the surface area is increased greatly. However, the space between adjacent “petals” of the ZnO nanoflowers is so small (~ 30 nm wide), as shown in the zoom-in view of Fig. 3a, that the following infiltration of the organic light absorber become very difficult. Figure 3b presents the top view of the ZnO nanocubes. Obviously, the ZnO nanocubes are uniform in size and the length of the side is about 150 nm. Besides, each ZnO nanocube is separated off from one another, which will influence the electron transport in solar cells, as described later in this article.

**Fig. 4:**
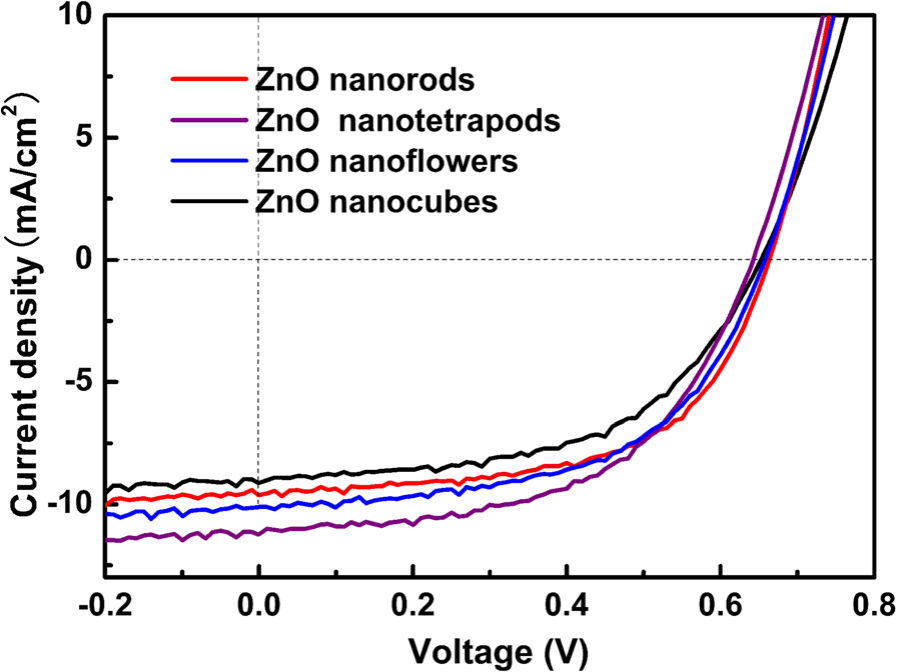
*J*-*V* characteristics of the organic bulk heterojunction solar cells with different ZnO nanostructures

Next, the four types of ZnO nanocrystals are introduced into the organic bulk heterojunction solar cells, as shown in Fig. 1. During the fabrication process, four solar cells were fabricated in each ITO substrate. Among which, if the maximum photon conversion efficiency (PCE) deviation is less than 3% in at least three solar cells with higher PCE values, then their performance parameters will be recorded. The highest PCE values in the records were adopted here for comparison. There, five samples were made for each example, among which, the PCE and other key parameters deviation for each example is less than 3%, thus the results are believable. The *J*-*V* characteristics of the solar cell devices with different ZnO nanocrystals under simulated sunlight were shown in Fig. 4, and the corresponding device performance is summarized in Table 1.

**Table 1:** The device performance of the organic bulk heterojunction solar cells

ZnO nanostructure	*J*_SC_ (mA/cm^2^)	*V*_OC_(*V*)	FF(%)	PCE(%)	*R*_series_ (Ω(cm^2^)
Nanorods	9.67	0.66	58	3.71	73.7
Nanotetrapods	11.24	0.65	55	3.96	71.4
Nanoflowers	10.2	0.66	55	3.69	93.8
Nanocubes	9.01	0.66	56	3.25	109.9

It can be seen that the ZnO nanotetrapod device shows a highest PCE of 3.96, followed by the ZnO nanorod and nanoflower device (3.71 and 3.69, respectively), and the ZnO nanocube device showed a lowest PCE of 3.25. The improvement in PCE arises from the higher short-circuit current density (*J*_SC_), while the open circuit voltage (*V*_OC_) of the four devices remains almost unchanged. The best performance of ZnO nanotetrapod device can be ascribed to the large surface area and proper space (~ 300 nm) between adjacent ZnO nanocrystals for the infiltration of the organic light absorber. The ZnO nanorod device suffers from relatively lower surface area, leading to lower dye loading and light harvesting, which will affect the charge extraction, and thus shows a lower *J*_SC_ compared with the ZnO nanotetrapod device [13]. The ZnO nanoflowers, as shown in Fig. 2c, d, has the largest surface area, but the corresponding device presents lower PCE compared with the ZnO nanotetrapod. Because the space (less than 50 nm) between adjacent “petals” of the ZnO nanoflowers is so close that the infiltration and the combination of the organic light absorber and the ZnO electron transport channel become very poor. As known, to achieve a higher ability of carrier transmission and exciton dissociation, better infiltration and more effective contact are essential. Hence, the ZnO nanoflower device suffers lower *J*_SC_, compared with the ZnO nanotetrapod. Besides the large surface area and proper space between adjacent ZnO nanocrystals for the infiltration of the organic light absorber, a short and continuous pathway for electron transport is also very important. For the ZnO nanocube device, as each ZnO nanocube is separated off from one another, the pathway for electron transport, which is interrupted by the grain boundary between adjacent nanocubes, is not continuous. As a result, the ZnO nanocube device presents the lowest *J*_SC_.

## Conclusions

In conclusion, we have synthesized various ZnO nanocrystals via a simple and cost-effective hydrothermal process. By tuning hydrothermal growth parameters, such as solution concentration, reaction temperature, and surfactant, ZnO nanorods, nanotetrapods, nanoflowers, and nanocubes have been obtained. These ZnO nanocrystals with different morphologies were further introduced into the active layer of organic bulk heterojunction solar cell as the electron transport channel. It was found that the device performance was closely related to the morphology of the ZnO nanocrystals. To improve the device performance, large surface area, proper space between adjacent ZnO nanocrystals, and a short and continuous pathway for electron transport, are essential.

## Abbreviations

HMTA: Hexamethylenetetramine
ITO: Indium-tin-oxide
*J*_SC_: Short-circuit current density
*J*-*V*: Current density-voltage
P3HT: Poly(3-hexylthiophene)
PCBM: [6]-Phenyl-C61-butyric acid methyl ester
PCE: Photon conversion efficiency
PEDOT: PSS: Poly(3,4-ethylene dioxythiophene):poly(styrene sulfonate)
PEI: Polyethylenimine
SEM: Field emission scanning electron microscopy
V_OC_: Open circuit voltage
